# Antiangiogenesis Therapy of Endometriosis Using PAMAM as a Gene Vector in a Noninvasive Animal Model

**DOI:** 10.1155/2014/546479

**Published:** 2014-06-24

**Authors:** Ningning Wang, Bin Liu, Lili Liang, Yanxin Wu, Hongzhe Xie, Jiaming Huang, Xu Guo, Jinfeng Tan, Xuejun Zhan, Yongdong Liu, Liantang Wang, Peiqi Ke

**Affiliations:** ^1^Department of Obstetrics and Gynecology, The First Affiliated Hospital of Sun Yat-sen University, 58th Zhongshan 2nd Road, Guangzhou 510080, China; ^2^Department of Pathology, The First Affiliated Hospital of Sun Yat-sen University, No. 58th Zhongshan 2nd Road, Guangzhou 510080, China

## Abstract

*Objective*. To evaluate the characteristics and antiangiogenic effects of endostatin-loaded PAMAM on endometriosis in a noninvasive animal model. *Materials and Methods*. A noninvasive animal model was established by injecting adenovirus-GFP transfected endometrial stromal and glandular epithelial cells subcutaneously into nude mice. Endostatin-loaded PAMAM was prepared and identified by transmission electron microscopy. For *in vitro* studies, the DNA protection and cytotoxicity of PAMAM were investigated and compared with Lipofectamine 2000. For *in vivo* study, endostatin-loaded PAMAM was injected into the noninvasive model and evaluated by continuously observing the fluorescent lesion, lesion weight, microvessel density and VEGF immunostaining. *Results*. Compared with Lipofectamine 2000, PAMAM and HC PAMAM-ES group, MC PAMAM-ES group and LC PAMAM-ES group demonstrated a better stromal cells protective such that MC PAMAM-ES group of CCK8 was 0.617 ± 0.122 at 24 hr and 0.668 ± 0.143 at 48 hr and LC PAMAM-ES group of CCK8 was 0.499 ± 0.103 at 24 hr and 0.610 ± 0.080 at 48 hr in stromal cells (*P* < 0.05) but similar cytotoxicity in glandular epithelial cells *in vitro*. After 16 hrs of digestion, DNA decreased slightly under the protection of PAMAM. Endostatin-loaded PAMAM of HD PAMAM-ES group and LD PAMAM-ES group inhibited the growth of the endometriotic lesion *in vivo* at days 15, 20, 25 and 30 detected by noninvasive observation after injecting one dose endostatin of various medicines into the endometrial lesion in each mouse on day 10 (*P* < 0.05) and confirmed by lesion weight at day 30 with HD PAMAM-ES group being 0.0104 ± 0.0077 g and LD PAMAM-ES group being 0.0140 ± 0.0097 g (*P* < 0.05). Immunohistochemistry results showed that endostatin-loaded PAMAM reduced the microvessel density 3.8 ± 2.4 especially in HD PAMAM-ES group in the lesion (*P* < 0.05). *Conclusion*. Endostatin-loaded PAMAM inhibits the development of endometriosis through an antiangiogenic mechanism and can be observed through the noninvasive endometriosis model.

## 1. Introduction

Endometriosis, defined as functioning endometrium outside the uterine cavity, is a common disease in women of reproductive age. Patients suffering from endometriosis may develop chronic pelvic pain, dysmenorrhea, dyspareunia, and infertility [1]. The prevalence of endometriosis has increased in recent years, while the etiology and mechanism have not been completely understood. Previous research shows that retrograde shedding [2], adherence and ectopic implantation [3, 4], and angiogenesis [5, 6] of the endometrium are three important steps in the development of endometriosis. In these processes, angiogenesis plays an important role in the formation of endometriosis because growing ectopic lesions need rich blood supply. Therefore, antiangiogenesis therapy is an important approach in the management of endometriosis.

In previous studies, endostatin has been identified as a useful and safe inhibitor for angiogenesis [1, 7–11]. However, as a protein reagent, endostatin can only function for a short time and thus is not suitable for a recurrent disease such as endometriosis. Therefore, we presumed that, a long-term drug delivery system, transferring endostatin gene into endometriotic lesions might be a better strategy for the treatment of endometriosis.

On the basis of our previous studies, the 20 *μ*g endostatin gene transduced by 65 *μ*g Lipofectamine 2000 inhibited the growth of endometriotic lesions with some stromal cells (ESCs) inhibited without any reproductive side effects in a nude mouse model [12]. Recently, a novel compound, polyamidoamine (PAMAM) dendrimers, has been certified as a nontoxic gene vector with high transduction efficiency [13]. However, the effect of PAMAM varied in different studies and has not been confirmed in the research on endometriosis.

To test the effect of the PAMAM-transduced endostatin gene on endometriosis, we applied the same noninvasive animal model established successfully by our research group [14]. In this model, we labeled endometrial stroma cells (ESCs) and grandular epithelial cells (EECs) separately with green fluorescence to form human endometriotic lesions by isolation-transfection-incubation procedure in nude mice and followed the fluorescent label* in vivo* noninvasively. This model helped us to repeatedly observe the lesion, and the application of the image analysis software helped us to study lesion changes quantitatively. At the same time, the accurate quantitative application of PAMAM and its effect are assessed* in vitro* and* in vivo.*

## 2. Materials and Methods

### 2.1. Endometrium Sample Collection

Samples of proliferative phase endometrium which are confirmed by histology were obtained from 16 women who received hysteroscopy for diagnostic purposes in our department between April and June 2009. All participants were aged 20–45 yrs (mean age 30), without internal complications and with regular menstrual cycles and no hormonal treatment for the last 3 months before surgery. Pieces of normal endometrium were obtained in surgery and immediately transferred into 4°C DMEM/F12 (Gibco, Carlsbad, CA, USA) supplemented with 1% penicillin and streptomycin (HyClone, Logan, UT, USA). An informed written consent was given by each patient before tissue collection. This protocol was approved by the First Affiliated Hospital of Sun Yat-sen University Ethical Review Committee.

### 2.2. Isolation and Primary Culture of ESCs and EECs

All experiments started within 1 hr after collection of the endometrium. The isolation of endometrial cells was in accordance with the methods stated in Ryan et al. [15] with some modification. In detail, endometrial tissue was washed 3 times with PBS, transferred to DMEM/F12 (Gibco, Carlsbad, CA, USA), supplemented with 1% penicillin and streptomycin (HyClone, Logan, UT, USA), and cut into 1–2 mm^3^ pieces. Then, the tissue was digested with 2 mg/mL collagenase I (Gibco, Carlsbad, CA, USA) at 37°C in 5% CO_2_ for 1 hr. After digestion, a sterile 150 *μ*m polyethylene mesh filter (Shen Yue instrument store, Guangzhou, China) was used to remove undigested debris, followed by a 45 *μ*m cell strainer to separate EECs from ESCs. The EECs were backwashed with DMEM/F12 from the 45-*μ*m cell strainer (Shen Yue instrument store, Guangzhou, China) onto the dish. After being centrifuged, the ESCs and EECs were resuspended with DMEM/F12 and 10% FCS then cultured in a 6-well plate.

### 2.3. Preparation and Identification of the Endostatin Plasmid Mixture

The preparation of the PAMAM-Es plasmid mixture was performed according to the Dendritech Company guidelines. Six PAMAM dendrimers were dissolved in water at a concentration of 1 mg/mL and stored at 4°C. The human recombined endostatin plasmid was stored at −20°C. When used, the PAMAM solution and the endostatin plasmid were both rewarmed in room temperature on the bench for 20 min and mixed together at a ratio of 3.25 *μ*g : 1 *μ*g (PAMAM : endostation). After 20 min of coincubation, the mixture was identified by JEM-2010HR transmission electron microscopy (JEOL, Tokyo, Japan).

As a traditional gene vector, Lipofectamine 2000 was taken to compare with PAMAM in both* in vitro* and* in vivo* studies. The preparation of the Lipofectamine-endostatin plasmid mixture (Lipo-Es) was in accordance with the routine of our laboratory. Briefly, Lipofectamine 2000 and endostatin plasmid were rewarmed in room temperature on the bench top for 20 min and mixed together at a ratio of 3.25 *μ*g : 1 *μ*g and cultured for another 20 min before injection.

### 2.4. *In Vitro* Studies on the DNA-Protection Effect and Cytotoxicity of PAMAM

To determine the protection of plasmid by PAMAM, 5 U of DNase I was added into a 50 *μ*L solution containing PAMAM-Es or Lipo-Es plasmid or naked plasmid. The primary plasmid concentration in each group was 0.200 mg/mL. OD260 values were detected by biophotometer after 1, 4, 8, and 16 hrs.

Cytotoxicity of PAMAM and PAMAM-Es was processed on primary cultural ESCs or EECs. Purified cells were cultured in 96-well plates with a density of 5000 cells/well for ESCs and 20 cells mass/well for EECs, respectively. Twenty-four hours after primary culture, the medium in the 96-well plates was discarded and 100 *μ*L of OptiMEM (Gibco BRL, Grand Island, NY, USA) was added into each well. Then, the PAMAM-Es mixture with various dilutions, the Lipo-Es mixture, and the PAMAM solution (shown in Table 1) were added into each group. After culturing for 24 hrs, 10 *μ*L of the CCK-8 reagent was added into each well, incubated in 37°C, 5% CO_2_ for 2 hrs, and detected by a Tecan biophotometer at 495 nm.

### 2.5. *In Vivo* Observation and Quantitative Analysis of GFP-Expressing Lesions in Nude Mice

According to our preliminary work [14], the isolation-transfection-incubation mixed cells injecting subcutaneous procedure was also performed in this experiment. Forty female nude mice (BALB/c), aged 6–8 weeks and weighing 17–21 g, were provided by the National Rodent Laboratory Animal Resources, Shanghai Branch (Shanghai, China). Two days before endometrial cell injection, a sterile 60-day release pellet, containing 1.7 mg of 17-beta E2 (Innovative Research of America, USA), was added to implant s.c. for every nude mice.

For* in vivo* imaging of GFP-expressing endometrial lesions, animals were put on a fluorescent stereomicroscope (SZX16, Olympus, Tokyo, Japan) equipped with a 470 nm filter. Images were recorded with an Olympus DP71 digital camera (Olympus, Tokyo, Japan) fixed with a 515 nm viewing filter. Each mouse was observed 6 times on days 5, 10, 15, 20, 25, and 30 after cells implantation.

Quantitative analysis was completed primarily according to Fortin et al. [16]. In short, at each time point, three fluorescent images were acquired for each mouse. Image software was used to identify the number and intensity of pixels corresponding to the spectral signature of GFP (present only in regions of interesting where there is a lesion) and the size of each lesion. If more than one lesion was present on a mouse, the calculated surface is the sum of each individual lesion.

To investigate whether the fluorescent area in the imaged lesion is representative of the actual lesion size, we used a traditional method to measure the length (*a*) and width (*b*) of the lesion with a vernier caliper and calculated the volume of the lesion with a well-recognized formula (*V* = (1/2)*a* × *b* ^2^). Then, a correlation test was applied to study the correlation between the volume of the lesion and the fluorescent area in the photo.

### 2.6. Treatment of Human Recombined Endostatin Plasmid Using PAMAM as a Vector

Forty nude mice were randomly divided into 5 groups according to their weight, lesion volumes, and fluorescent pixel numbers on days 5 and 10: (1) HD PAMAM-Es group: 20 *μ*g Es/65 *μ*g PAMAM; (2) LD PAMAM-Es group: 10 *μ*g Es/32.5 *μ*g PAMAM; (3) Lipofectamine-Es group: 20 *μ*Es/65 *μ*g Lipofectamine; (4) PAMAM group: 65 *μ*g PAMAM; (5) PBS group: same volume PBS. Each group received different treatments (shown in Table 2) by injecting one dose of various medicines into the endometrial lesion in each mouse on day 10, just after the second noninvasive observation.

Three approaches were used to evaluate the antiangiogenesis effects of PAMAM-Es. First, the variation in fluorescent areas of the lesions before and after treatment was calculated and compared among each group. Second, volume changes among the 5 groups were analyzed. Third, after 30 days of* in vivo* observation, mice were sacrificed and lesions in different groups were excised and weighed with an electronic balance.

### 2.7. Evaluation of Antiangiogenesis Efficiency of PAMAM-Endostatin

After being weighed, the lesions were then fixed in 4% PFA for 24 hours at room temperature and embedded in paraffin. Sections of 5 *μ*m were first stained with hematoxylin and eosin to evaluate the lesions' viability and quality. Microvessel density (MVD) and VEGF expression of the lesion were determined by immunohistochemistry staining using horseradish peroxidase detection system (Zhongshan, Beijing, China) with polyclonal rabbit anti-CD31 antibody (dilution of 1 : 250, Abcam) and monoclonal rabbit anti-human VEGF antibody (dilution of 1 : 250, Abcam). The slides were counterstained with hematoxylin.

Two blinded observers examined the tissue with a microscope (IX71, Olympus, Tokyo, Japan). For the MVD calculations, the regions with the highest microvessel density (hot spots) were scanned at low magnification (×40) as described by Weidner [17] and counted at a ×400 magnification in a blinded fashion. For each slide, microvessels were counted twice in 5 different high magnifications and the average was used as the final value.

For VEGF analysis, slides were first scanned at low magnification (×40), and five fields of the immunostained sections were randomly chosen for histomorphometry at ×400 magnification. A semiquantitative evaluation of immunohistochemical staining for VEGF was performed according to the method described by Donnez et al. [18], involving the analysis of the distribution and the intensity of staining within the endothelium and glandular epithelium or stroma. The histologic scores (*H*) for VEGF were calculated using the formula *H* = Σ*P*_*i*_, where *i* is the intensity ranging from 0 (negative cells) to 3 (deeply staining cells) and *P* is the percentage of staining cells for each given *i*, with *P* values of 1, 2, 3, 4, and 5 indicating <15%, 15–50%, 50–85%, >85%, and 100% positive-staining cells, respectively. The staining result was expressed as the mean ± standard deviation.

### 2.8. Statistics

Data were analyzed by SPSS 13.0 software. For the cytotoxicity study* in vitro*, OD values of various treatments were described as the mean ± SD and analyzed by one-way ANOVA. For noninvasive* in vivo* studies, the corelationship between fluorescent pixel numbers and the volumes of each lesion was tested. A two-way ANOVA analysis (main effect: group and time; interaction: group and time) was applied to compare lesion fluorescent pixel numbers among groups. For the invasive study, lesion weight, MVD count, and VEGF histology scores were compared among groups using one-way ANOVA. In this study, all the ANOVA tests were followed by a post hoc Bonferroni test to detect differences between groups. Two-tailed values of *P* < 0.05 were considered statistically significant.

## 3. Results

### 3.1. The Characteristics of ESCs and EECs in Primary Culture and after Viral Transfection

**Figure 1:**
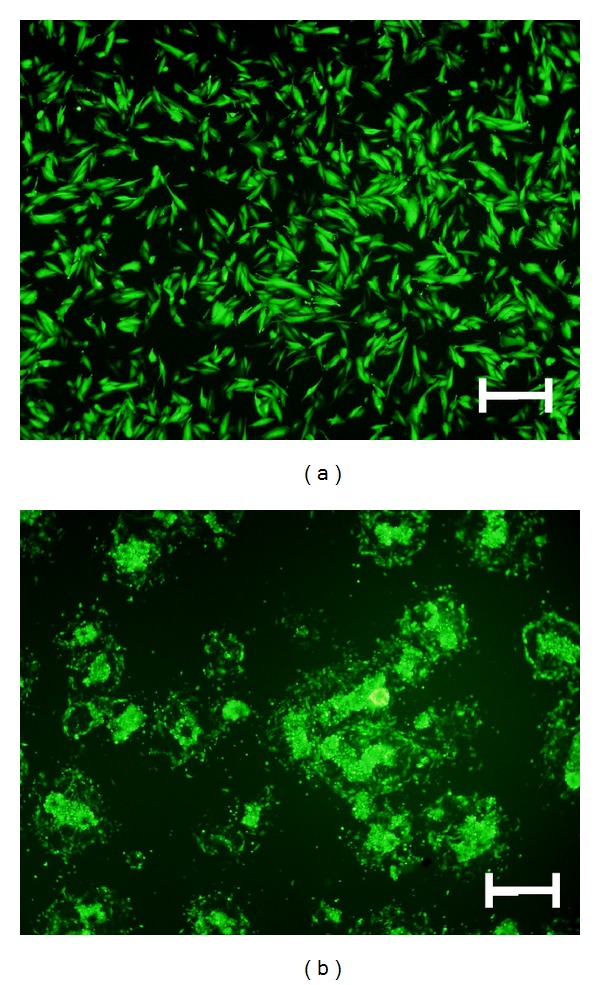
Primary cultural stromal cells (a) and glandular epithelial cell masses (b) expressed green fluorescent after adenovirus-eGFP transfection for 18 h (100x, Bar = 200 *μ*m).

ESCs attached to the 6-well plate 12 hrs after placement began to express GFP. The intensity of fluorescence increased until 18 hrs and was maintained at a high level (Figure 1). According to our previous result, after 18 hrs of incubation, both ESCs and EECs had high GFP positive rates and low apoptosis rates [14]. Thus, ESCs and EECs were harvested 18 hrs after transfection and collected to build a noninvasive animal model.

### 3.2. Morphology, DNA Protection Effect, and Cytotoxicity of PAMAM-Es

**Figure 2:**
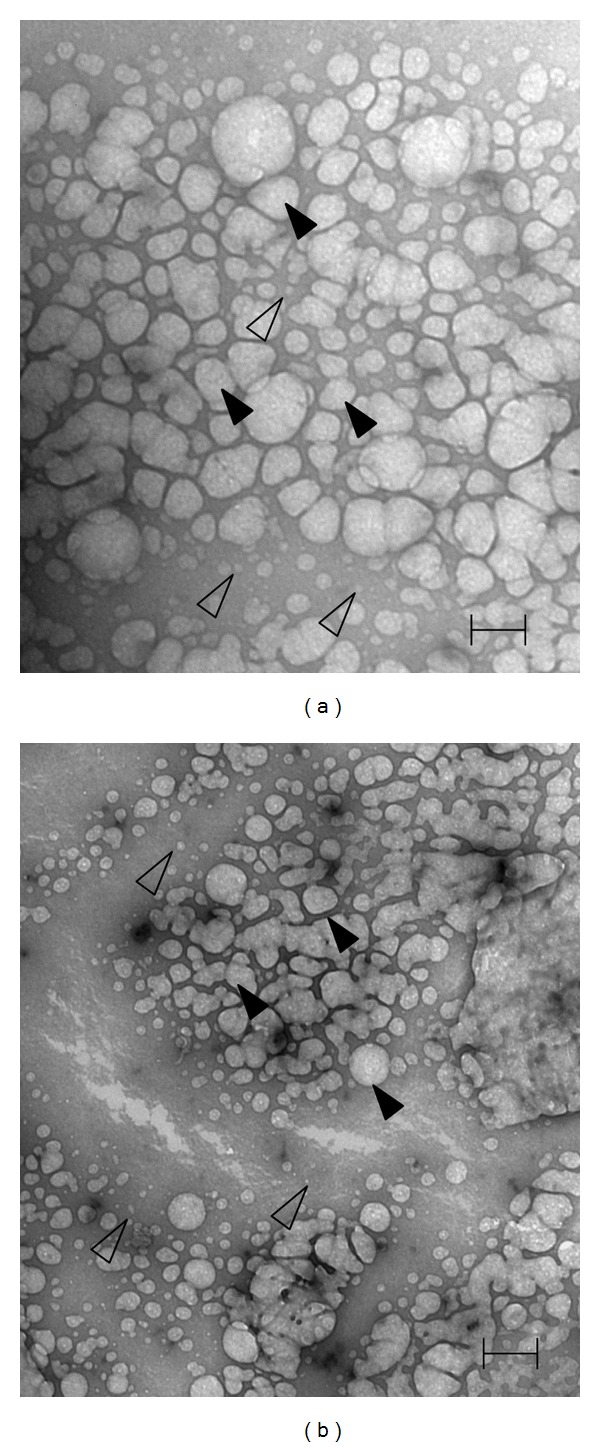
Structure of PAMAM and PAMAM-Es was similar under transmission electron microscopy (37000x, BAR = 200 nm). (a) Morphology of PAMAM under TEM. (b) Morphology of PAMAM-Es under transmission electron microscopy. Open arrows: PAMAM monomer (diameter: 5–10 nm); close arrows: PAMAM polymerize compound (diameter: 50–200 nm).

The morphology of PAMAM and PAMAM-Es was detected by transmission electron microscopy (Figure 2). The diameter of the PAMAM monomer ranged from 5 to 10 nm, consistent with the description of the product (5.4 nm). The PAMAM monomer polymerized and formed compounds with diameters ranging from 50 to 200 nm. No differences were found between PAMAM and PAMAM-Es in morphology under transmission electron microscopy.

**Figure 3:**
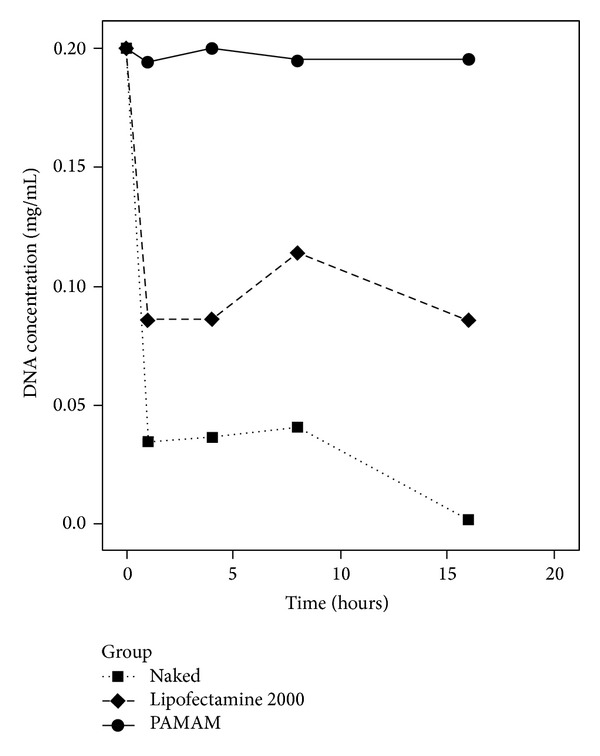
DNA concentration after digestion by DNase I (mg/mL).

In the study on the DNA protective effects, as shown in Figure 3, the DNA concentration of the PAMAM-Es mixture was much higher than the Lipofectamine-Es mixture and the naked endostatin plasmid after digestion by DNase I. After 16 hrs of digestion, the naked plasmid had almost broken down, and the plasmid protected by Lipofectamine reduced to approximately half of the primary concentration, while DNA decreased slightly under the protection of PAMAM.

To analyze cytotoxicity in various reagents to ESCs and EECs, OD495 absorbance, representing live cell activity, was compared among each group via one-way ANOVA. As shown in Table 3, toxicity levels among 6 types of reagents to ESCs, rather than to EECs, had statistically significant differences at both 24 and 48 hrs. The post hoc Bonferroni test showed that, when compared with the control group, high concentrations of PAMAM-Es, Lipo-Es, and PAMAM groups significantly inhibited the activity and growth of ESCs, while the inhibition of ESCs with moderate concentrations of PAMAM-Es and low concentrations of PAMAM-Es groups was not significant and demonstrated a better stromal cells protective.

### 3.3. *In Vivo* Observation and Therapeutic Effects of PAMAM-Endostatin

**Figure 4:**
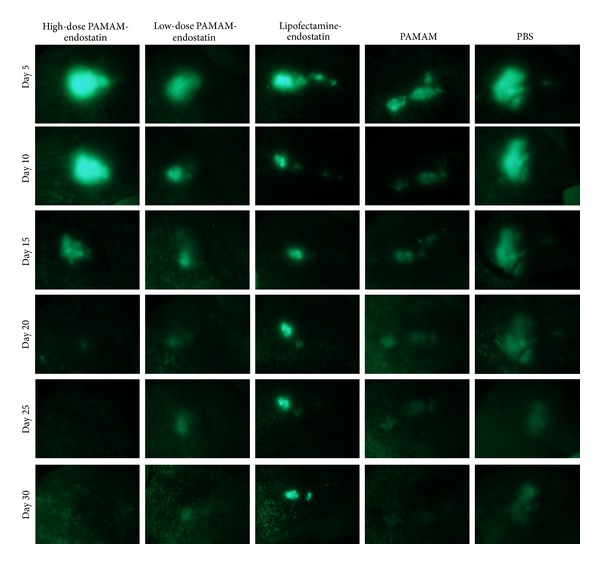
*In vivo* observation by fluorescent microscopy of nude mice in each group.

**Figure 5:**
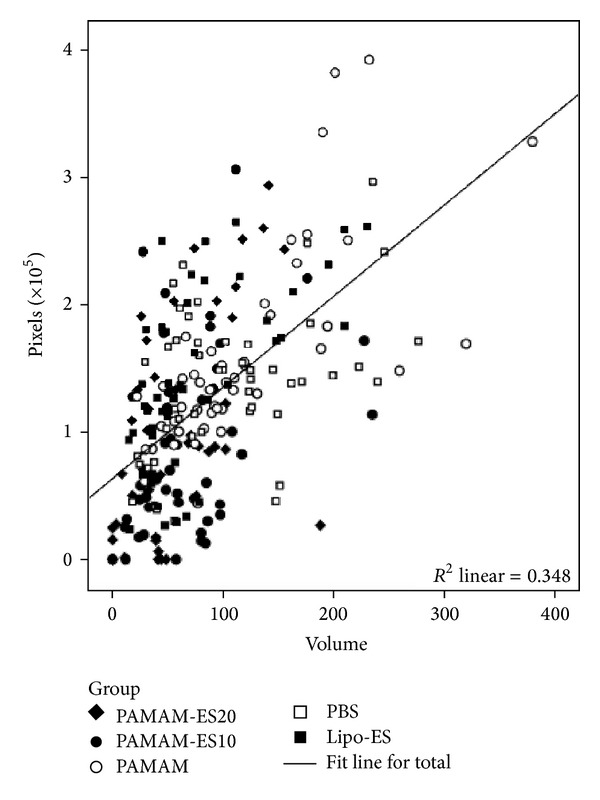
Correlation between fluorescent pixel number and lesion volume.

Using fluorescent microscopy, positive lesions on the models are easily detected (Figure 4). All fluorescent images were quantitatively analyzed. To identify the fluorescent area in the photos, we studied the corelationship between the fluorescent area in the image and the volume of the same lesion, as measured by the vernier caliper and calculated with a formula. The correlation test illustrated that, when combining data from five groups, there is a statistically significant positive correlation between the fluorescent pixel numbers and the lesion volumes (Figure 5, with a *R* = 0.590 and *P* < 0.001). This result demonstrated that the fluorescent area was representative of the lesion size in the noninvasive* in vivo* study. Therefore, we used the quantitatively analyzed fluorescent area to study the therapeutic effects of PAMAM-Es on endometriosis.

First, to evaluate the homogeneity of pretreatment states among groups, lesion volumes and fluorescent areas on days 5 and 10 were compared by one-way ANOVA. The results showed that no significant differences existed between the lesion volumes and the fluorescent areas among groups.

Then, we compared the inhibition of PAMAM-Es to endometriotic lesions with Lipofectamine-Es, PAMAM, and PBS controls. Two-way ANOVA tests showed that there was a statistically significant difference among the groups (Table 4). The post hoc Bonferroni test demonstrated that high-dose and low-dose PAMAM-Es reduced the size of the lesions compared with other reagent groups.

**Figure 6:**
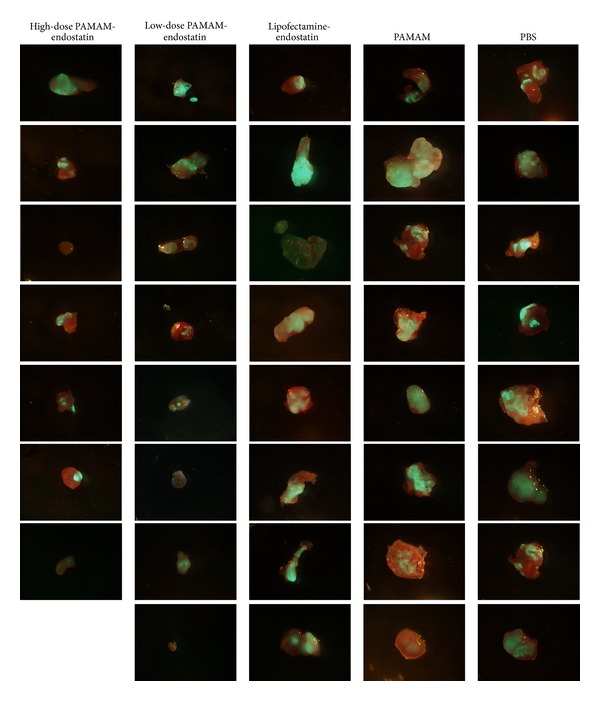
Fluorescent lesions observed after necropsy (combined by fluorescent imaging and white light picture).

After 30 days of noninvasive observation, mice were sacrificed and the lesions were collected (Figure 6) and weighed. The one-way ANOVA test showed that the tumor burden among each group had a statistically significant difference. In the post hoc Bonferroni test, the HD PAMAM-Es group had a statistically significant lower lesion weight than PAMAM and PBS controls and the LD PAMAM-Es group had a statistically significant lower lesion weight than PBS controls (Table 5).

### 3.4. Evaluation of the Antiangiogenesis Efficiency of PAMAM-Es

**Figure 7:**
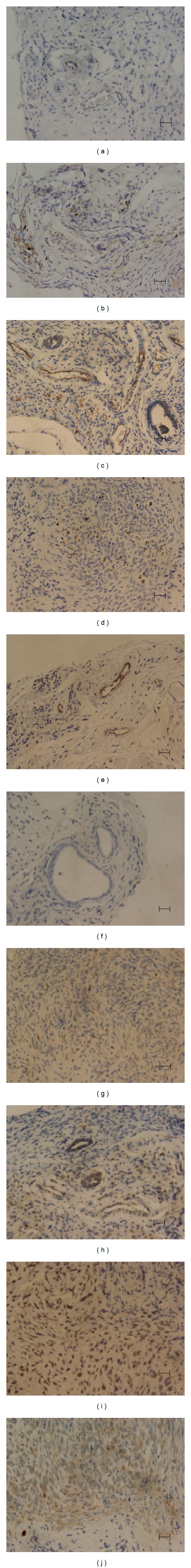
CD31 and VEGF expression in endometrial lesions in nude mice (400x, Bar = 100 *μ*m) (a–e): CD31 expression in microvessels in the implanting lesions ((a) HD Pamam-Es; (b) LD PAMAM-Es; (c) Lipofectamine-Es; (d) PAMAM; (e) PBS); (f–j): VEGF expression in cells of the implanting lesions ((f) HD Pamam-Es; (g) LD PAMAM-Es; (h) Lipofectamine-Es; (i) PAMAM; (j) PBS).

To investigate whether PAMAM-Es inhibited endometriotic lesions through an antiangiogenesis mechanism, two well-recognized angiogenesis biomarkers, CD31 and VEGF, were detected by immunohistochemistry (Figure 7) on every lesion and compared among the groups. There was a statistically significant difference in MVD, calculated as CD31-stained vessels per ×400 magnification, among the 5 groups in the ANOVA test (*P* = 0.023). HD PAMAM-Es group showed a greater antiangiogenesis effect than both the PAMAM and PBS groups in the post hoc Bonferroni test (Table 6). The ANOVA test found no difference in the VEGF *H* score among the 5 groups (*P* = 0.092) (Table 6).

## 4. Discussion

Although several approaches can be applied, there is no ideal therapy method for endometriosis. Hormonal treatment has substantial side effects, whereas it does not remove the ectopic lesion radically. Conservative surgery can excise the ectopic lesion, but the disease recurs in part of patients. Radical surgery can directly affect reproducibility. Thus, new methods should be investigated to treat endometriosis [19]. Considering the characteristic of endometriosis, an ideal reagent should have followed these features: (1) reducing or removing the ectopic lesion; (2) reducing side effects, especially to the reproductive system; (3) preventing recurrence.

Endostatin is considered the most effective inhibitor of microvessel growth [1] and has been used to treat endometriosis [1, 7–11, 20, 21]. Our previous results [12] showed that 20 *μ*g Lipofectamine mediated endostatin for lesion injection could significantly reduce the volume of the ectopic lesion by destroying established vessels after 21 days treatment or inhibiting angiogenesis factor-VEGF mRNA at day 3 treatment in the lesion. However, the effect of Lipofectamine mediated endostatin can last a period but still existed ESCs inhibited potentially. Common lesions in nude mice should wait near 28 days enough to form a 4 mm diameter lesion to observe the treatment result. To resolve this problem, we attempted to change noninvasive model and more effective vector in the experiment treatment.

The green fluorescent human endometriosis lesion model which was supported on isolation-transfection-incubation procedure by injecting GFP-Adenovirus transfected human ESCs and EECs subcutaneously into nude mice was continued to be used for noninvasively evaluating the effect of various treatments [14]. Except for high transfection rate and low apoptosis rate, there were several other advantages. First, GFP labeling enabled us to repeatedly observe the lesion in a noninvasive manner; thus, changes in lesions after treatment could be detected directly rather than from necropsy, and continuous data rather than endpoint information could be more accurately analyzed. Second, injection of separated cells rather than a whole tissue increased the reliability of the results. Researchers usually implanted whole pieces of transfected and nontransfected endometrium to build an endometriosis model. In these cases, although the same pieces of tissue were used in each host animal, the exact number of cells injected was unknown. However, in our study, the number of injected cells was controlled and the baseline of each host mouse was the same.

To the best of our knowledge, it was the first study applying polyamidoamine (PAMAM) dendrimers as a gene vector in the antiangiogenesis therapy of endometriosis. We primarily tested the cytotoxicity of the PAMAM-Es compound on ESCs and EECs* in vitro*. CCK-8 studies demonstrated that stromal cells were sensitive to high concentrations (HC) of PAMAM-Es, PAMAM, and Lipofectamine 2000-Es but not by moderate (MC) or low concentrations (LC) of PAMAM-Es demonstrating such that MC PAMAM-Es and LC PAMAM-Es reagent could be more safely used for treatment than Lipofectamine 2000 that we have used before. On the DNA protective effects, DNA decreased slightly under the protection of PAMAM. On the basis of* in vitro* experiment, the dose of endostatin used in the MC PAMAM-Es group was 0.00001 *μ*g per stromal cell* in vitro*, which is calculated to be applied with HD PAMAM-Es group (20 *μ*g Es/2∗10^6^ ESCs)* in vivo. In vivo* noninvasive observations in the nude mice model illustrated that PAMAM-Es with a dose-dependent effect had been more effective on inhibiting the growth of endometriotic lesions and avoiding ESCs affected than the Lipofectamine Es.

To uncover whether PAMAM-Es inhibited the growth of endometriotic lesions because of its antiangiogenesis effect, we employed the fluorescent pixel number which was positive correlation to lesion volume and the immunohistochemistry to detect the microvessel density of the lesion. The results demonstrated further that 20 *μ*g PAMAM-Es significantly directly reduced the microvessel density leading to a decrease in fluorescent pixel number. These noninvasive observing results support our previous research not only on the endostatin gene therapy but also on PAMAM mediated for the management of endometriosis. There are primarily two strategies for antiangiogenesis therapy. One is to prevent angiogenesis in the newly developing lesion; the other is to inhibit vascular growth in the established lesion. Therefore, in experimental endometriosis, antiangiogenesis reagents were used before or at the time of transplantation as preventive measures or after lesion formation as treatment. Eggermont et al. demonstrated that newly developing vessels were usually established between 5 and 8 days after graft implantation [22]. Thus, our data suggested that, after transduction and expression, endostatin could also inhibit established vessels in the endometriotic lesion after 10 days implantation.

Further, VEGF is considered a pivotal angiogenic factor by inducing endothelial cell-specific mitogenic and vascular permeability activities [20, 23]. Immunostaining showed that PAMAM-Es did not induce a reduction directly in VEGF expression after 20 days treatment as same as the lipofectamine Es but short effect should be confirmed in the future experiment.

In summary, compared with the traditional gene carrier Lipofectamine* in vitro* and* in vivo,* study in the present research demonstrates that PAMAM is an ideal vector in gene therapy for the treatment of endometriosis. Endostatin-loaded PAMAM inhibits the development of endometriosis through an antiangiogenic mechanism and can be observed through the green fluorescent endometriosis model.

## 5. Conclusions

Endostatin-loaded PAMAM inhibits the development of endometriosis through an antiangiogenic mechanism and can be observed through the noninvasive endometriosis model.

## Figures and Tables

**Table 1:** Medicines used in cytotoxicity test.

Group	Plasmid (*μ*g/mL)	Vector (*μ*g/mL)	Number of wells
(1) HC PAMAM-Es	10	32.5	6
(2) MC PAMAM-Es	1	3.25	6
(3) LC PAMAM-Es	0.1	0.325	6
(4) Lipofectamine-Es	10	32.5	6
(5) PAMAM	—	32.5	6
(6) Blank control	—	—	6

**Table 2:** Medicines used in treatment of animal model.

Group	Endostatin plasmid (*μ*g)	Vector (*μ*g)	Number of mice
(1) HD PAMAM-Es	20	65	8
(2) LD PAMAM-Es	10	32.5	8
(3) Lipofectamine-Es	20	65	8
(4) PAMAM	—	65	8
(5) PBS	—	65 uL	8

**Table 3:** Cytotoxicity test of ESCs and EECs by CCK-8 Kit (absorbance, mean ± S.D).

	Stromal cell		Glandular epithelial cell	
	24 h	48 h	24 h	48 h
Control	0.625 ± 0.155	0.648 ± 0.117	0.409 ± 0.252	0.233 ± 0.114
HC PAMAM-Es	0.118 ± 0.019^a^	0.088 ± 0.007^a^	0.385 ± 0.218	0.325 ± 0.060
MC PAMAM-Es	0.617 ± 0.122	0.668 ± 0.143	0.395 ± 0.156	0.308 ± 0.192
LC PAMAM-Es	0.499 ± 0.103	0.610 ± 0.080	0.337 ± 0.167	0.290 ± 0.071
Lipo-Es	0.202 ± 0.081^a^	0.136 ± 0.049^a^	0.347 ± 0.171	0.255 ± 0.063
PAMAM	0.167 ± 0.083^a^	0.096 ± 0.018^a^	0.328 ± 0.138	0.265 ± 0.054
*F*	31.444	71.95	0.193	0.651
*P*	0.000	0.000	0.963	0.663

^a^Statistic significant difference compared with control group.

**Table 4:** Area of fluorescent lesion in treatment and control groups at different time point (pixel).

Group	Day 5	Day 10	Day 15	Day 20	Day 25	Day 30
(1) HD PAMAM-Es^a^	223104 ± 60101	146451 ± 54606	83812 ± 52424	59549 ± 49634	33301 ± 36204	29676 ± 36394
(2) LD PAMAM-Es^b^	179970 ± 64910	111880 ± 59332	91058 ± 73949	55692 ± 46240	46290 ± 39019	43015 ± 41680
(3) Lipofectamine-ES^a,b^	194664 ± 62033	163435 ± 56293	113506 ± 50036	117513 ± 62957	120533 ± 71327	131120 ± 71257
(4) PAMAM^a,b^	200465 ± 58021	154130 ± 60652	128977 ± 62570	138480 ± 85150	160714 ± 105618	161172 ± 92100
(5) PBS^a,b^	184576 ± 57799	159941 ± 78005	130626 ± 50700	126370 ± 58317	111729 ± 44760	123874 ± 62956

^a^Statistic significant difference between Group 1 and Group 3, Group 1 and Group 4, and Group 1 and Group 5 by Bonferroni test. ^b^Statistic significant difference between Group 2 and Group 3, Group 2 and Group 4, and Group 2 and Group 5 by Bonferroni test. (Injecting one dose of various medicines into the endometrial lesion in each group mouse on day 10.)

**Table 5:** Lesion weight of different groups (gram, mean ± SD).

Group	Lesion weight
(1) HD PAMAM-Es^a^	0.0104 ± 0.0077
(2) LD PAMAM-Es^b^	0.0140 ± 0.0097
(3) Lipofectamine-Es	0.0253 ± 0.0158
(4) PAMAM^a^	0.0350 ± 0.0245
(5) PBS^a,b^	0.0378 ± 0.0170

^a^Statistic significant difference between Group 1 and Group 4 and Group 1 and Group 5 by Bonferroni test. ^b^Statistic significant difference between Group 2 and Group 5.

**Table 6:** Numbers of MVD (CD31-stained vessels per ×400 magnification) and VEGF *H* score (per ×400 magnification) in endometriosis lesions in nude mice (mean ± SD).

Group	MVD	VEGF *H* score
(1) HD PAMAM-Es	3.8 ± 2.4^a^	3.8 ± 3.1
(2) LD PAMAM-Es	10.5 ± 3.9	4.7 ± 2.6
(3) Lipofectamine-Es	11.4 ± 4.7	5.2 ± 3.2
(4) PAMAM	11.9 ± 6.7^a^	7.0 ± 3.1
(5) PBS	12.1 ± 4.3^a^	8.1 ± 2.3

^a^Statistic significant difference between Group 1 and Group 4 and Group 5.
